# Multicenter study on hemorrhagic risk of heparin bridging therapy for periendoscopic thromboprophylaxis

**DOI:** 10.1186/s12876-015-0315-1

**Published:** 2015-07-28

**Authors:** Mio Matsumoto, Katsuhiro Mabe, Momoko Tsuda, Masayoshi Ono, Saori Omori, Masakazu Takahashi, Takeshi Yoshida, Shoko Ono, Manabu Nakagawa, Soichi Nakagawa, Yuichi Shimizu, Takahiko Kudo, Naoya Sakamoto, Mototsugu Kato

**Affiliations:** 1 Department of Gastroenterology, Sapporo Medical Center NTT EC, 〒060-0061 South 1 West 15, Chuo-ku, Sapporo, Hokkaido Japan; 2 Division of Endoscopy, Hokkaido University Hospital, 〒060-0814 North 14, West 5, Kita-ku, Sapporo, Hokkaido Japan; 3 Department of Gastroenterology, Hokkaido University Graduate School of Medicine, 〒060-0814 North 14, West 7, Kita-ku, Sapporo, Hokkaido Japan; 4 Department of Gastroenterology, Sapporo City General Hospital, 〒060-8604 North 11, West 13, Chuo-ku, Sapporo, Hokkaido Japan

**Keywords:** Endoscopic treatment, Post-procedural bleeding, Thrombosis, Antithrombotic therapy

## Abstract

**Background:**

For endoscopic interventions, heparin bridging therapy is recommended in patients who are at high risk from interruption of antithrombotic therapy. Although heparin bridging has been reported to be effective in preventing thrombosis, several reports have raised concerns about increased risk of bleeding. The aim of this study was to clarify complications of hepari  bridging therapy in therapeutic endoscopy.

**Methods:**

A nationwide multicenter survey using questionnaire was performed about patients undergoing therapeutic endoscopy with heparin bridging. Patients who underwent therapeutic endoscopy without heparin bridging therapy were considered as controls. Compliance scores of heparin bridging therapy guideline were employed, and association was analyzed between the score and occurrence of post-procedural bleeding.

**Results:**

The incidence of post-procedural bleeding was significantly higher (13.5 %, 33/245) in the heparin group compared with the control group (2.7 %, 299/11102)(p < 0.001). Thrombosis occurred in 1 patient each in the two groups. In the heparin group, post-procedural bleeding was more likely to be delayed bleeding. Dose adjustment of heparin was a significant factor contributing to bleeding. The compliance score of heparin bridging therapy guideline was significantly higher in those who suffered bleeding.

**Conclusions:**

Heparin bridging therapy significantly increased the risk of post-procedural bleeding compared with the control. The bleeding risk was associated with greater adherence with guidelines for heparin bridging therapy.

## Background

Antithrombotic therapy with antiplatelet or anticoagulant agents is widely used for the primary and secondary prophylaxis of cardiovascular and cerebrovascular disorders, and patients using oral antithrombotic agents have been increasing worldwide.

Conventionally, guidelines recommended temporary interruption of antithrombotic therapy for endoscopic interventions [1]. Recent guidelines emphasize the importance of thrombosis prophylaxis and recommend not to interrupt antithrombotic therapy as long as possible [2, 3].

Heparin bridging therapy(HBT) has been a strategy conventionally used for patients requiring interruption of antithrombotic therapy but at high risk from the interruption of antithrombotic therapy, whether anticoagulants or antiplatelet agents. According to recent guidelines, for those receiving antiplatelet therapy, HBT is not recommended and antiplatelet therapy should be continued with low-dose aspirin during the peri-procedural period [2, 3]. For those receiving warfarin, however, its interruption has been reported to lead to potentially fatal serious thrombosis in 0.6 % to 1.0 % of cases [4–6]. Thus, HBT remains recommended for patients on oral anticoagulant therapy who are at high risk from interruption of the therapy [2, 3, 7–9].

While some manuscripts reported the effectiveness of HBT in preventing thrombosis, others describe an increased risk of bleeding during HBT [10–12].

The present study was conducted to clarify complications of HBT in patients undergoing therapeutic endoscopy, which is classified among endoscopic procedures posing a high risk of bleeding.

## Methods

**Fig. 1:**
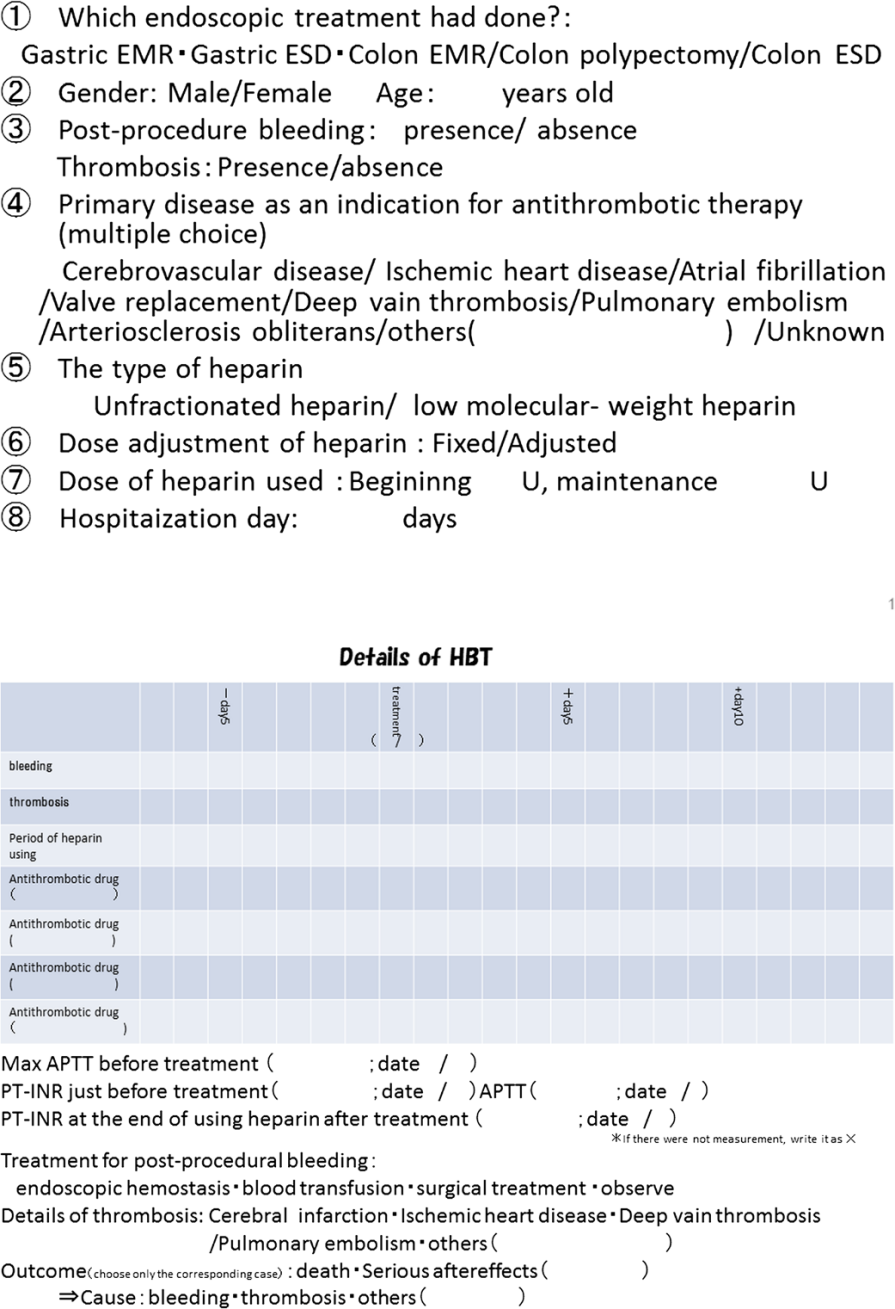
Questionnaires

A nationwide multicenter survey with questionnaire was performed in Japan, about patients who received HBT in therapeutic endoscopy (Fig. 1).

This survey had been performed in accordance with the Declaration of Helsinki and had been approved in the Ethics Committee of Hokkaido University Hospital (IRB No. 012–0093).

### Patients and therapeutic endoscopy

Subjects were successive patients who underwent a therapeutic gastroscopy or colonoscopy using HBT at participating institutions between April 2009 and March 2011. Evaluated anticoagulants were limited to warfarin and dabigatran which were available oral anticoagulants during the study period in Japan.

Inclusion criteria was following endoscopic interventions; gastric endoscopic mucosal resection (EMR) and endoscopic submucosal dissection(ESD), and colon polypectomy, EMR and ESD.

The interventions for esophagus, duodenum and small intestine, percutaneous endoscopic gastrostomy(PEG), and billio-pancreatic therapy were excluded.

The manner of each endoscopic treatment followed the guideline of Japan Gastroenterological Endoscopy Society.

### Survey items

The following data were collected by retrospectively reviewing the medical records; age, sex, specific oral antithrombotic drugs (anticoagulants including warfarin, dabigatran, antiplatelet drugs including aspirin, clopidogrel, and cilostazole), primary disease as an indication for antithrombotic therapy, type of heparin used, any dose adjustment of heparin, any coagulation tests and their data around the time of heparin use, and timing of the resumption of chronic antithrombotic therapy. Dose adjustment of heparin was defined as administration of heparin at an adjusted dose according to the activated partial thromboplastin time (APTT). No dose adjustment of heparin referred to administration of heparin at a fixed dose irrespective of the APTT.

Data on complications were collected in terms of the following: presence or absence of any complications (post-procedural bleeding and thrombosis) occurring between the interruption of antithrombotic therapy and two weeks after the therapeutic endoscopy, timing of its occurrence, presence or absence of treatment for the complication, and details of treatment if any. Post-procedural bleeding was defined as bleeding that occurred after therapeutic endoscopy and met any the following criteria: (1) apparent post-procedural hematemesis or melena requiring emergency endoscopic intervention or open surgery; (2) Forrest grade Ia or Ib bleeding noted on a follow-up endoscopic evaluation; or (3) bleeding requiring blood transfusion.

Controls were patients who underwent therapeutic endoscopy without HBT at the same participating medical institutions during the same period as above. Data on controls were collected in terms of the number of therapeutic endoscopies, and the numbers of patients with post-procedural bleeding and thrombosis.

### Participation institutions

Among all the coaching institutions of Japan Gastroenterological Endoscopy Society that are known to perform therapeutic endoscopy, 22 were contacted and 17 institutions agreed to participate in the study.

### Endpoints

The primary endpoints were the incidences of post-procedural bleeding and thrombosis among patients who underwent therapeutic endoscopy on HBT. The secondary endpoints were factors contributing to bleeding, timing of bleeding, and association between heparin bridging guideline compliance scores and occurrence of post-procedural bleeding.

### Compliance scores

Compliance scores of HBT guideline were employed to measure whether appropriate bridging was performed. For the scoring, in patients who received heparin bridging during interruption of their chronic anticoagulant therapy, the following items were assessed according to guideline recommendations:

(1) the duration of pre-procedural interruption of warfarin therapy, 3–5 days; (2) APTT during heparin bridging, 1.5- to 2-fold higher than normal; (3) timing of post-procedural resumption of warfarin therapy, within 2 days of the procedure; and (4) prothrombin time-International normalized ratio (PT-INR) at post-procedural discontinuation of heparin bridging, >therapeutic range.

The score was “1” if the guideline recommendation was appropriately followed, “0” if the guideline recommendation was not checked, and “-1” if the treatment was against the guideline recommendation. The ranges of guideline recommendations for these items were defined as follows on the basis of the 2002 Guidelines by the American Society of Gastrointestinal Endoscopy (ASGE) [13] and the 2009 Guidelines by the Japanese Circulation Society.

### Statistical analysis

The chi-square test was used to compare the heparin bridging group and the control group. The chi-square test and the logistic regression analysis were used to assess the association between bleeding and various factors. Factors with a p-value of <0.5 on the univariate analysis were further assessed using the multivariate logistic regression analysis. A p-value of <0.05 was considered as statistically significant. All statistical calculations were performed using the software JMP® 11 (SAS Institute Inc., Cary, NC, USA).

## Results

### Incidences of post-procedural bleeding and thrombosis

According to the questionnaire responses by the 13 participating medical institutions in Japan, a total of 245 patients received HBT in the setting of therapeutic endoscopy, specifically for upper gastrointestinal treatment in 171 patients and lower gastrointestinal treatment in 74 patients. The controls were 4488 patients who received upper gastrointestinal treatment and 6614 patients who received lower gastrointestinal treatment without HBT.

In the HBT group, the mean age was 73.4 ± 8.5 years. Heparin was a bridge to antiplatelet medication in 111 patients, because this is retrospective study including the period which was also recommended HBT for antiplatelet medication by guideline. The type of heparin used for the bridge was standard unfractionated heparin in 239 patients and low-molecular-weight heparin in 6 patients (Table 1).

**Table 1:** Baseline characteristics of study patients

	Upper GI treatment	Lower GI treatment	Total
HBT patients (number)	171	74	245
Mean age (mean ± S.D.)	74.1 ± 8.6	71.7 ± 8.0	73.4 ± 8.5
Sex (M: F)	142:29	57:17	199:46
Therapy before HBT			
anticoagulants	91	43	134
antiplatelets	80	31	111
Antithrobmotic			
Single	80	32	112
Multiple	91	42	133
Condition requiring antithrobmotic			
Atrial fibrillation	46	22	68
Ischemic heart disease	53	33	86
Deep vein thrombosis	7	1	8
Valve replacement	9	8	17
Arteriosclerosis obliterans	5	4	9
Cerebrovascular disease	46	18	64
Type of heparin used			
Unfractionated heparin	171	68	239
LMWH	0	6	6

HBT: heparin bridge therapy, GI: gastrointestinal, LMWH: low molecular- weight heparin. A patient could be counted under more than one condition

Post-procedural bleeding occurred in 13.5 % (33/245) patients in the HBT group and 2.7 % (299/11102) in the control group, with the incidence significantly higher in the HBT group (p < 0.001). In the case which received heparin as a bridge to anticoagulant medication, post-procedural bleeding occurred in 15.7 % (21/134), to antiplatelet medication, post-procedural bleeding occurred in 10.8 %(12/111). As for the incidence of post-procedural bleeding by site of endoscopic treatment, that among patients who received upper gastrointestinal treatment was 14.1 % (25/171) in the HBT group and 4.5 % (204/4488) in the control group, and that among patients who received lower gastrointestinal treatment was 10.8 % (8/74) and 1.4 % (95/6614) respectively, thus showing similar results to the above (p < 0.001) (Table 2). Post-procedural bleeding was treated with blood transfusion in 12.1 % (4/33) in the HBT group and 10.7 % (32/299) in the control group, with no difference between the two groups. Post-procedural bleeding was manageable with either endoscopic intervention or conservative treatment in all patients in the HBT group, but required open surgery in 2 of 299 patients in the control group. No deaths occurred in either group.

**Table 2:** Incidence of post-procedural bleeding by site of treatment

	HBT group	Control group	p-value
Total	13.5 %	2.7 %	<0.001
	(33/245)	(299/11102)	
Upper gastrointestinal tract	14.1 %	4.5 %	<0.001
	(25/171)	(204/4488)	
Lower gastrointestinal tract	10.8 %	1.4 %	<0.001
	(8/74)	(95/6614)	

Thrombosis occurred in 1 patient each in the HBT group and the control group who both received upper gastrointestinal treatment, with no significant difference between the two groups. The patient in the　HBT group who suffered thrombosis was a 79-year-old male. He had been receiving oral low-dose aspirin and clopidogrel because of ischemic heart disease. For a planned gastric ESD, both drugs were discontinued and administration of heparin was started, and 2 days later cerebral infarction occurred. After conservative treatment, the cerebral infarction improved, and the patient was once discharged from the hospital 10 days after the onset of cerebral infarction. On a later day he was re-hospitalized and underwent ESD on heparin bridging.

The patient in the control group who suffered thrombosis was a 72-year-old male. He had been receiving oral low-dose aspirin alone after coronary artery bypass grafting for angina pectoris. He concurrently had atrial fibrillation but was not taking warfarin. Four days after discontinuation of the low-dose aspirin therapy, cerebral infarction occurred. After conservative treatment, the cerebral infarction improved, and ESD was performed 24 days after the onset of cerebral infarction. Both patients had no further thrombosis at the second interruption of their long-term antithrombotic therapy.

### Used drugs at the time of bleeding

**Fig. 2:**
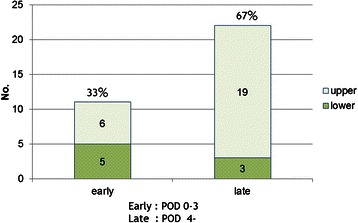
Early vs. Delayed bleeding. Early, within 3 postprocedural days; Delayed, day 4–14 after postprocedural day. POD = post operative day

The timing of the occurrence of post-procedural bleeding was more likely post-procedural day 4 or later, compared with the previously reported distribution of the timing of bleeding after therapeutic endoscopy [14–17]. When bleeding was classified according to the timing of onset into two types, i.e., early bleeding (defined as bleeding within 3 post-operative days) and late bleeding (defined as bleeding from post-operative day 4 to 14), early bleeding accounted for 33 % and late bleeding for 67 %, showing that late bleeding was more common (Fig. 2).

**Fig. 3:**
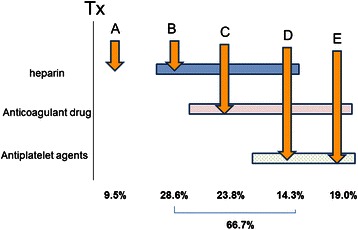
Antithrombotic therapy status at bleeding. Among 21 patients who received heparin as a bridge to anticoagulant medication and experienced post-procedural bleeding, the timing of bleeding was analyzed with the timing classified into five categories from A to E according to the antithrombotic therapy status at bleeding. A, before post-procedural resumption of heparin; B, after resumption of heparin but before resumption of oral antithrombotic therapy; C, during concomitant use of heparin and an anticoagulant; D, during concomitant use of three drugs of heparin, an anticoagulant, and an antiplatelet drug; E, after the end of heparin bridging and during oral antithrombotic therapy. The percentages at the bottom indicate the proportions of patients who had bleeding during the time periods

Among 21 patients who received heparin as a bridge to anticoagulant medication and experienced post-procedural bleeding, the timing of bleeding was analyzed with the timing classified into five categories from A to E (Fig. 3). Specifically, the timing “A” was defined as before post-procedural resumption of heparin, the timing “B” as after resumption of heparin but before resumption of chronic oral antithrombotic therapy, the timing “C” as during concomitant use of heparin and anticoagulant medication, the timing “D” as during concomitant use of three drugs of heparin, anticoagulant medication, and antiplatelet medication, and the timing “E” as after the end of heparin bridging and during chronic oral antithrombotic therapy.

Bleeding during the timing “A” accounted for 9.5 % (2/21), “B” for 28.6 % (6/21), “C” for 23.8 % (5/21), “D” for 14.3 % (3/21), and “E” for 19.0 % (4/21). In 1 patient (4.8 %), bleeding occurred after resumption of chronic oral anticoagulant therapy without resumption of heparin bridging. Combined timing of “B + C + D,” i.e., bleeding during use of heparin, accounted for 66.7 %. Bleeding occurred most commonly during concomitant use of heparin and anticoagulant medication.

### Factors contributing to bleeding

As for factors contributing to bleeding, the univariate analysis was performed. Next, factors with a p-value of <0.5 on the univariate analysis assessed by the multivariate logistic regression analysis(age, the type of antithrombotic drugs, number of antithrombotic drugs, and dose adjustment of heparin for bridging). Both the univariate analysis and the multivariate analysis showed that post-procedural bleeding was significantly more common in patients given bridging therapy with dose adjustment of heparin according to the APTT measured (Table 3).

**Table 3:** The univariate and multivariate analysis for post-procedural bleeding

	Bleeding	No bleeding	Univariate (p value)	Multivariate OR	95 % CI	Multivariate (p value)
Age	72.2 ± 8.6	73.6 ± 8.5	0.40	0.26	0.01-9.30	0.45
Sex (M/F)	29/4	170/42	0.71			
Anticoagulants vs. Antiplatelet drugs^a^	21:12	114:98	0.28	1.36	0.61-3.17	0.45
Number of antithrombotic drugs Multiple vs. single^a^	21:12	112:100	0.25	1.88	0.84-4.38	0.12
Type of heparin	32:1	207:5	0.81			
Unfractionated heparin vs. LMWH						
Dose adjustment of HBT	17:16	36:176	<0.001	5.06	2.29-11.3	<0.001
Adjusted vs. Fixed^a^						
Timing of antithrombotic resumption Appropriate vs. Inappropriate	16:17	119:93	0.51			

OR: odds ratio, CI: confidence interval, LMWH: low molecular- weight heparin, HBT: heparin bridge therapy

^a^Control in multivariate analysis

To examine the influence of heparin resumed after the procedure, similar analyses were performed in a subgroup of patients on long-term warfarin therapy, but again dose adjustment of heparin was a significant factor.

### Compliance score of HBT guideline

**Fig. 4:**
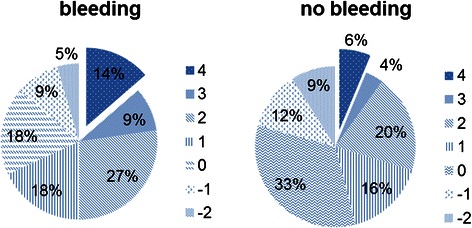
Compliance score of HBT guideline. Distribution of compliance scores of HBT guideline is shown for patients with bleeding and patients without bleeding

Compliance scores of heparin bridging guideline were calculated among patients who received heparin as a bridge to anticoagulant medication. The highest possible total score in each patient was 4. HBT was guideline-compliant in terms of all assessed items in 14 % of those who suffered bleeding, compared with as few as 6 % of those who did not suffer bleeding (Fig. 4). The mean score was 1.52 in those with bleeding and 0.62 in those without bleeding, with the score significantly higher in patients who suffered bleeding (p = 0.01). These results demonstrated that guideline-compliant HBT was associated with increased bleeding.

## Discussion

More and more patients are now on chronic oral antithrombotic therapy, and antithrombotic management in the setting of therapeutic endoscopy is an important issue. Many recent guidelines emphasize the importance of thrombosis prophylaxis and recommend not to interrupt any antithrombotic therapy in the setting of procedures with a low bleeding risk such as endoscopic examination and biopsy [2, 3]. Although Asians are considered more likely to suffer bleeding compared with Westerners, and the duration of antithrombotic therapy interruption was described to be likely longer in Eastern countries than in Western countries [18], in 2012 the Japan Gastroenterological Endoscopy Society updated its guideline on perioperative interruption of antithrombotic therapy [19] and made it in line with Western guidelines. Compared with antiplatelet drugs, anticoagulants have a higher bleeding risk and also their interruption poses a higher thrombotic risk [20–23], so the use of warfarin requires measurements of blood coagulability (PT-INR) and the coagulability level needs to be within the therapeutic range. For therapeutic endoscopy as a procedure with a high bleeding risk, however, interruption of antithrombotic therapy for a certain period with bridging using heparin is recommended, while data have been scarce on bleeding with HBT.

This survey revealed that the incidence of post-procedural bleeding was significantly higher in the HBT group compared with the control group. Bleeding was common during post-procedural concomitant use of warfarin and heparin. Compared with typical bleeding after therapeutic endoscopy, delayed post-procedural bleeding was more common, for which caution is warranted. Dose adjustment of heparin was a factor contributing to bleeding, and the incidence of bleeding was higher with guideline-compliant HBT. In other words, the data suggest that the risk of bleeding is higher with proper HBT with maintained coagulability. Thus, HBT requires further evaluations. The incidence of thrombosis did not differ between those given and not given HBT, and clinicians should keep in mind that the risk of thrombosis is not zero even with HBT.

While Yoshio et al. documented increased occurrence of delayed bleeding after gastric ESD on HBT [10], the present survey in the setting of therapeutic endoscopy, including both upper gastrointestinal and lower gastrointestinal treatments, demonstrated that the incidence of post-procedural bleeding was significantly higher with HBT than without it. And this finding in the HBT group of this survey was consistent with a previously published report that described an increased incidence of delayed post-procedural bleeding among patients on oral antithrombotic therapy [24].

The present survey revealed that the methods of HBT varied between different medical institutions, and fully guideline-compliant HBT was performed in as few as 8 % of the patients. Inappropriate methods of HBT may not avoid thrombosis. Thrombosis with heparin was reported bridging only in 1 patient in this survey. However, in light of published literature on fatal thrombosis during interruption of antithrombotic therapy for endoscopic intervention [25], appropriate HBT would be desirable from the viewpoint of thrombosis prophylaxis.

However, HBT guideline is not for bleeding but avoiding thrombosis. The present survey also revealed that the incidence of bleeding was higher with guideline-compliant HBT. Also, bleeding was common during concomitant use of heparin and antithrombotic medication. In light of published reports describing a lower incidence of bleeding with continuation of warfarin than with HBT [11, 12] and the anticoagulants is not risk factor of post endoscopic surgery bleeding [26], the bleeding risk of HBT requires reassessment. Another literature report states that new anticoagulants such as dabigatran and rivaroxaban do not require bridging [27]. Interruption of anticoagulant therapy needs further detailed evaluations, including the use of new anticoagulants as well as continuation of warfarin within the therapeutic range.

Previous HBT guideline had recommended even for antiplatelet drugs, not just for the anticoagulant drugs. This is the retrospective survey including the period of previous guideline, so many of the patients received heparin as a bridge to antiplatelet medication. However bleeding was common even in those given heparin as a bridge to antiplatelet medication. Recent guidelines recommend continuation of aspirin or cilostazol therapy during the peri-procedural period, and do not recommend heparin as a bridge to antiplatelet medication. However, for patients on both oral anticoagulant and antiplatelet drugs in combination, further studies are required to determine whether HBT should be performed, and if HBT is performed, whether concomitant antiplatelet medication should be given during HBT.

One of the limitation of this study was that this was a retrospective questionnaire survey. So no background data could be collected for patients in the control group, and no comparison was allowed for factors contributing to bleeding between the HBT group and the control group. Another limitation was that not all endscopic interventions enrolled.

Despite this limitation, this nationwide multicenter survey in Japan revealed a higher incidence of bleeding after therapeutic endoscopy during HBT. The present survey is also meaningful in that it is the first study assessing bleeding in both the upper and lower gastrointestinal tracts after endoscopy on HBT.

In conclusion, HBT had significant risk of the incidence of post-procedural bleeding compared with control. The bleeding risk was associated with greater adherence with guidelines for HBT. Further studies are required for the use of heparin bridging in those who are at high risk from interruption of antithrombotic therapy.

## Conclusions

Heparin bridging therapy significantly increased the risk of post-procedural bleeding compared with the control. The bleeding risk was associated with greater adherence with guidelines for heparin bridging therapy.

## Abbreviations

HBT: Heparin bridging therapy
EMR: Endoscopic mucosal resectio
ESD: endoscopic submucosal dissection
PEG: percutaneous endoscopic gastrostomy
APTT: the activated partial thromboplastin time
ASGE: the American Society of Gastrointestinal Endoscopy
